# The Adipocyte–Macrophage Relationship in Cancer: A Potential Target for Antioxidant Therapy

**DOI:** 10.3390/antiox12010126

**Published:** 2023-01-04

**Authors:** Sofía Sanhueza, Layla Simón, Mariana Cifuentes, Andrew F. G. Quest

**Affiliations:** 1 Cellular Communication Laboratory, Center for Studies on Exercise, Metabolism and Cancer (CEMC), Institute of Biomedical Sciences (ICBM), Faculty of Medicine, University of Chile, Santiago 8380492, Chile; 2 Advanced Center for Chronic Diseases (ACCDiS), Faculty of Medicine, University of Chile, Santiago 8380492, Chile; 3 Laboratory of Obesity and Metabolism in Geriatrics and Adults (OMEGA), Institute of Nutrition and Food Technology (INTA), Universidad de Chile, Santiago 7830490, Chile; 4 Escuela de Nutrición y Dietética, Universidad Finis Terrae, Santiago 7501015, Chile

**Keywords:** adipose tissue, obesity, inflammation, antioxidants, endocrine signaling, cancer cells, tumor microenvironment, paracrine signaling

## Abstract

Obesity has emerged as a major public health concern with a staggering 39% worldwide prevalence as of 2021. Given the magnitude of the problem and considering its association with chronic low-grade systemic inflammation, it does not come as a surprise that obesity is now considered one of the major risk factors for the development of several chronic diseases, such as diabetes, cardiovascular problems, and cancer. Adipose tissue dysfunction in obesity has taken center stage in understanding how changes in its components, particularly adipocytes and macrophages, participate in such processes. In this review, we will initially focus on how changes in adipose tissue upon excess fat accumulation generate endocrine signals that promote cancer development. Moreover, the tumor microenvironment or stroma, which is also critical in cancer development, contains macrophages and adipocytes, which, in reciprocal paracrine communication with cancer cells, generate relevant signals. We will discuss how paracrine signaling in the tumor microenvironment between cancer cells, macrophages, and adipocytes favors cancer development and progression. Finally, as reactive oxygen species participate in many of these signaling pathways, we will summarize the information available on how antioxidants can limit the effects of endocrine and paracrine signaling due to dysfunctional adipose tissue components in obesity.

## 1. Introduction

### 1.1. Obesity: A Major Worldwide Health Problem

Obesity is characterized by an excessive accumulation of white adipose tissue that elevates health risks and is considered a severe public health problem. The already epidemic proportions continue to grow throughout the world and are of great concern given that numerous diseases are either triggered or aggravated by this disorder. Indeed, the COVID-19 pandemic revealed that people with obesity were at greater risk for unfavorable outcomes related to disease severity and death [1]. Obesity is a complex multifactorial disease with etiological components that range from molecular to social factors, and given the well-established connection to the development of multiple chronic diseases, it is of utmost importance to better understand the molecular mechanisms involved in order to prevent or revert related comorbidities. 

### 1.2. White Adipose Tissue Dysfunction and Disease Development: Role of Macrophages and Adipocytes

For a long time, adipose tissue was considered a simple energy reservoir; however, after the first reports in the 1990s on its ability to secrete numerous key regulatory factors, this tissue has come to be recognized as a major endocrine organ [2,3]. Besides the secretion of regulatory proteins, adipose tissue communicates with other organs and aids in maintaining homeostasis through the release of many different mediators, including lipids, metabolites, noncoding RNAs, and extracellular vesicles (EVs) [4]. Importantly, obesity is frequently characterized by adipose tissue dysfunction; it is often associated with infiltration by macrophages, ensuing changes that result in the liberation of many proinflammatory factors, insulin resistance, and impaired lipid metabolism. Local changes in adipose tissue are transmitted to various organs via this altered secretome and thereby contribute to deleterious forms of organ crosstalk typically associated with the development of chronic diseases, such as nonalcoholic fatty liver disease, cardiovascular disease, type 2 diabetes, and cancer. 

Adipose tissue expansion due to excessive energy intake is accompanied by numerous changes in tissue function and composition [5]. Particularly, dysfunctional fat is characterized by inflammation, driven mainly by the infiltration of proinflammatory CD11c-expressing M1-polarized macrophages. Once the tissue has been infiltrated, M1 macrophages and adipocytes establish a complex reciprocal communication network involving cytokines, chemokines, and microRNA-containing exosomes, among others, that precipitate functional dysregulation [6]. In addition, one key hallmark of adipose tissue inflammation is the presence of histological markers, termed crown-like structures (CLS), generated by proinflammatory macrophages surrounding dead or dying adipocytes [7]. 

In the context of this review, the fact that obesity has been associated with the development of several types of cancer, including endometrial, breast, ovarian, prostate, liver, gallbladder, kidney, and colon cancer, is of particular interest. Indeed, the aforementioned inflammatory changes that adipose tissue undergoes in obesity have been mechanistically linked to multiple aspects of cancer development.

### 1.3. Obesity-Associated Cancer

A 2016 report that analyzed the results from over 1000 epidemiological studies [8] determined that there is sufficient evidence in humans to conclude that the absence of excess body fat lowers the risk for thirteen sites/types of cancer, among which are esophagus adenocarcinoma, colon and rectum, liver, pancreas, breast (postmenopausal), and corpus uteri cancer. The study of the mechanisms involved in this relationship, which may be relevant to all cancers and/or particular to specific cases, is extremely important for identifying therapeutic targets and developing novel treatment strategies. However, given the wide variety of alterations involved in obesity-related pathophysiology on the one hand and the complexity of the mechanisms that are altered during different carcinogenic processes on the other, it is difficult to address all aspects in a single model. For instance, the development of some types of cancer may be more specifically favored by obesity-related alterations in sex hormone metabolism while others are stimulated by enhanced insulin and insulin growth factor signaling, altered adipokine levels, or elevated proinflammatory and/or oxidative status [9,10,11]. 

Interestingly, adipocytes in obesity suffer several subcellular/metabolic alterations, such as mitochondrial dysfunction and endoplasmic reticulum (ER) stress. These changes result in an increased production of reactive oxygen species (ROS) as a consequence of excess food consumption and the ensuing overload of nutrients that need to be processed [12,13,14,15]. In turn, such constantly elevated ROS production is expected to further damage cell components and threaten cell viability, exacerbating the aforementioned formation of CLS, thereby generating a microenvironment favorable for tumor progression due to its effects on cell proliferation, survival, invasion, and metastasis [16]. In addition, obesity leads to an excess of adipose tissue, which in turn favors inflammation, dyslipidemia, hyperglycemia, and, consequently, oxidative stress. For instance, obesity promotes oxidative stress and favors tumor development by inducing DNA damage and genomic instability. Oxidative stress is further increased by mitochondrial alterations and potentiated by obesity-induced chronic inflammation. Moreover, obesity-induced dyslipidemia promotes lipid peroxidation and DNA mutations. Finally, obesity-induced hyperglycemia increases insulin signaling, glycolytic pathway activity, oxidative stress, and DNA alterations (reviewed in [17,18]).

### 1.4. Tumor Microenvironment: Focus on Adipocytes and Macrophages

As mentioned above, adipose tissue in obesity may undergo several structural, metabolic, and functional changes that create a proinflammatory and -oxidative milieu within the tissue microenvironment. These local changes involving cell–cell and cell–extracellular matrix crosstalk are thought to be particularly relevant in tumors that are in close proximity to adipose tissue, as is the case for breast cancer [19]. 

Multiple players in the obese adipose tissue microenvironment promote insulin resistance and elevate local insulin levels, which then can enhance phosphoinositide 3-kinase (PI3K) signaling and promote tumorigenesis [20,21]. Moreover, increased vascular endothelial growth factor (VEGF) production associated with adipose extracellular matrix remodeling likely facilitates tumor angiogenesis [22]. This matrix remodeling together with interstitial fibrosis in mammary adipose tissue has been shown to promote breast tumorigenesis by altering mammary epithelial cell malignancy and matrix mechanics [23]. In addition, factors secreted from adipose tissue that are elevated in obesity, such as leptin, visfatin, or those contained in EVs, have been shown to promote processes associated with tumor development, such as angiogenesis, invasion, viability, metalloproteinase activation, and ROS production, and do so by elevating ERK and PI3K/Akt signaling among other mechanisms [24,25,26].

### 1.5. Adipose Derangements and the Tumor Microenvironment as Potential Therapeutic Targets

**Figure 1:**
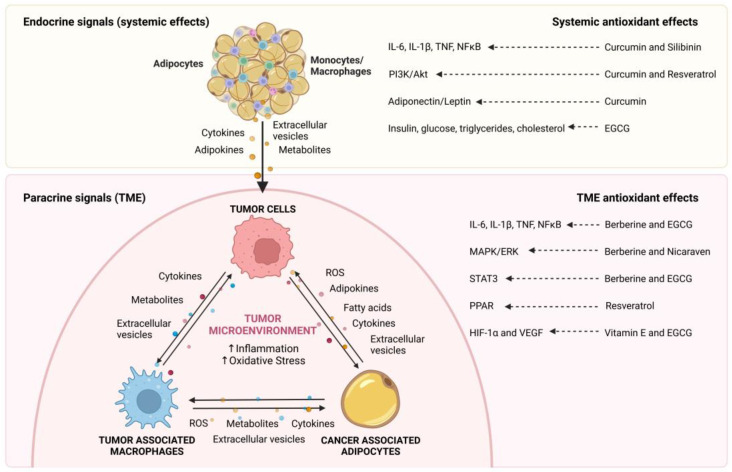
Endocrine and paracrine communication between adipocytes, macrophages, and cancer cells. Obesity-induced changes in the adipose tissue result in increased expression of cytokines, adipokines, metabolites, and extracellular vesicles (EVs) that influence cancer cell behavior and promote tumor development. In addition, in the tumor microenvironment (TME), adipocytes and macrophages are key components that interact with cancer cells by secreting a variety of signaling molecules, including, adipokines, cytokines, metabolites, fatty acids and EVs, which increase local inflammation and oxidative stress to generate a favorable microenvironment for tumor progression. Some antioxidants discussed in Section 5 and the pathways they control are also indicated.

Many of the proposed mechanisms linking obesity to cancer development involve well-known adipose tissue derangements that occur during excess weight gain. Consequently, therapeutic approaches aimed at decreasing adipose dysfunction are promising in order to uncouple these events. In particular, supplementation with natural or synthetic antioxidants is one of these strategies. For instance, obesity-induced metabolic disorders in rat models have been prevented or ameliorated by treatments with natural antioxidants, such as rutin [27], which also suppressed the formation of CLS and macrophage ROS production. Another study showed that dietary supplementation with oleuropein (an abundant phenolic compound in olives) in diet-induced obese mice decreased the levels of the angiogenic factor VEGF-A and suppressed the induction of solid tumor growth as well as lymph node metastasis [28]. In addition, the natural polyphenolic compound resveratrol has been shown to reduce or prevent many cancer-inducing effects related to the hyperglycemic conditions frequently observed in obesity, such as cell proliferation, ROS production, signaling cascades involved in tumor migration and invasion (ERK and p38 MAPK) as well as enhanced expression of factors relevant to cell migration, invasion, and metastasis [29]. Moreover, treatment of Glut1-overexpressing macrophages from diet-induced obese mice with N-acetylcysteine to quench ROS abolished the upregulation of the proinflammatory plasminogen activator inhibitor 1 (PAI-1) [30]. These few examples highlight the utility of antioxidants as potential therapeutic tools to prevent obesity-induced inflammation in cancer development. In the following sections, we will review the currently available information relating to endocrine and paracrine communication between adipose tissue and macrophages with a focus on their importance in tumor growth and the potential of different therapeutic approaches using antioxidants for delaying or preventing cancer development. The aspects that will be covered in this review are graphically summarized in Figure 1.

## 2. Endocrine Communication between Adipose Tissue and the Tumor Microenvironment

Obesity is associated with an increased risk of developing several types of cancer due to, among other mechanisms, chronic inflammation and metabolic dysregulation. Inflammation-related cytokines, such as interleukin-6 (IL-6) and C-reactive protein (CRP), as well as changes in metabolism-related adipokines, such as adiponectin and leptin, have been shown to reflect a proinflammatory microenvironment that promotes tumor development and progression. Patients with a normal weight tend to have higher levels of the anti-inflammatory and insulin-sensitizing adipokine adiponectin while those who are overweight or obese have higher leptin, IL-6 and CRP levels. In addition, elevated IL-6 is associated with increased cancer mortality in patients with obesity [31]. In the following paragraphs, we will focus on the mechanisms connecting adipose tissue to the tumor microenvironment as summarized in Table 1. 

Obese CLS+ tissue donors showed increased liver fat deposition and circulating tumor necrosis factor-alpha (TNFα) levels as well as insulin resistance, which are all important systemic consequences of disrupted adipose tissue homeostasis. Pathway analysis of differentially expressed genes in adipose tissue revealed the upregulation of various molecules belonging to the proinflammatory nuclear factor kappa B (NF-κB) signaling pathway in CLS+ explants [7]. Overweight and obese breast cancer patients develop CLS+ and adipose tissue inflammation, which is associated with markers of metabolic derangements, such as increased lipid, glucose and CRP levels in serum [42]. Moreover, breast cancer patients with adipose inflammation have increased insulin, glucose, leptin, triglycerides, CRP, and IL-6 levels as well as decreased HDL and adiponectin levels. Furthermore, obesity, metabolic syndrome and the circulation of proinflammatory molecules are associated with poorer prognosis in breast cancer patients. Indeed, adipose tissue inflammation reduces the distant recurrence-free survival of these patients [43].

In lung cancer, there is also a strong association between overweight/obesity, lipid alterations, and oxidative stress. Indeed, the total cholesterol/HDL ratio, triglyceride levels, lipid peroxidation and oxidative stress are increased in lung cancer patients compared to healthy people. Moreover, these alterations increase in lung cancer patients who are overweight and obese. These data suggest that obesity could enhance lung cancer-induced systemic changes, such as lipid and redox imbalance [47].

A high-fat diet (HFD) increases the myeloid-derived suppressor cell (MDSC) fraction, a heterogeneous population of cells of myeloid origin that mediates the suppression of T cells and contributes to the inhibition of immune responses during cancer [48,50]. In addition, a HFD enhances the M2/M1 macrophage ratio and accelerates prostate cancer tumor growth via IL-6/STAT3 signaling [48]. Furthermore, adipose tissue-derived factors observed in mice on a HFD stimulate the progression of prostate cancer via endocrine and paracrine mechanisms. For instance, HFD-induced obese mice showed increases in VEGF, IL-6, chemokine ligand 2 (CCL2), and C-X-C motif chemokine ligand 1/2/13 (CXCL1/2/13), which promote angiogenesis and the infiltration of immune cells that favor tumor development and metastasis [49].

Interestingly, statistical modeling of microRNA-mediated gene regulation identified common interactions in obesity, liver and uterine cancers. For instance, the upregulation of genes encoding for proteins involved in key metabolic processes, such as fatty acid and arachidonic acid metabolism, as well as signaling pathways related to cell growth and inflammation, are observed in all cases [35]. Furthermore, in endometrial cancer patients, body mass index (BMI), leptin, IL-6 and oxidative stress correlate with pathogenesis and poor prognosis [36]. Likewise, in breast cancer, several factors secreted by adipose tissue augment cancer cell aggressiveness. For instance, leptin secreted by adipose stromal/stem cells isolated from women with obesity enhances the expression of epithelial-to-mesenchymal transition (EMT) and metastasis-related genes, such as serpine 1, matrix metalloproteinase 2 (MMP-2) and IL-6 [37]. In addition, the reciprocal relationship between adipocytes, immune cells and cancer cells induces a systemically immunosuppressive macroenvironment that promotes breast cancer development. Moreover, breast cancer cells implanted in a mouse model of diet-induced obesity modified the visceral adipose tissue, spleen and tumor microenvironment in order to create an immunosuppressive milieu that promoted cancer development. The characteristic increase in the M1/M2 macrophage ratio reported in the visceral adipose tissue in obesity was completely reversed in tumor-bearing mice, resulting in a predominantly M2-polarized profile typically observed in the tumor microenvironment. Moreover, Tregs were decreased in obese mice, but increased in visceral adipose tissue in the presence of tumors. Altogether, these results suggest the existence of a regulatory feedback mechanism between the growing tumor and adipose tissue, which generates local and systemic immunosuppressive conditions that promote tumor development [38].

In addition to the aforementioned mechanisms, there is a link between obesity and breast cancer that includes a connection between adipocytes, endothelial and immune cells. The obese tumor microenvironment recruits macrophages that activate the NOD-like receptor 4 (NLRC4) inflammasome, leading to interleukin-1β (IL-1β) activation. IL-1β promotes breast cancer progression by increasing adipocyte VEGF secretion and angiogenesis [39]. Moreover, angiopoietin-like 4 (ANGPTL4), a known proangiogenic factor in cancer, is induced by IL-1β from adipocytes in a manner dependent on NF-κB and MAPK. This mechanism is further stimulated by hypoxia. In this way, adipocyte-secreted ANGPTL4 promoted angiogenesis and breast cancer progression in obese mice [40]. Furthermore, factors secreted by adipose tissue from HFD-induced obese mice and the fat tissue of female patients with obesity led to the upregulation of genes involved in inflammation and lipid metabolism, such as PPARα, IL-1β and ANGPTL4 in triple-negative breast cancer cells. Moreover, focal adhesion kinase (FAK) activation increased in these cancer cells, resulting in elevated lipid metabolism, wound healing, proliferation, and invasion [41].

As discussed in the previous paragraphs of this section, obesity and inflammation promote not only postmenopausal breast cancer progression but also metastasis. Adipocyte progenitors (CD45-CD34^+^) in xenograft and breast cancer models secrete granulocyte-macrophage colony-stimulating factor (GM-CSF) and matrix metalloproteinase 9 (MMP-9), thereby inducing immunosuppression, intratumor vascularization, as well as local and metastatic tumor growth [44]. Moreover, obesity increases lung neutrophilia and breast cancer metastasis to the lung in a GM-CSF and IL-5-dependent manner [45]. In addition, obesity induces the expression of sphingosine-1-phosphate (S1P), a bioactive sphingolipid mediator of breast cancer pathogenesis. Obesity enhances the expression of sphingosine kinase 1 (SphK1), the enzyme that produces S1P and its receptor S1PR1. Indeed, obesity-induced breast tumors increase SphK1 and secrete S1P, which promotes macrophage recruitment through S1PR1 in lung premetastatic niches. Macrophages secrete S1P, IL-6 and TNF*α*, thereby promoting breast cancer metastasis [46].

Regarding the role of specific proinflammatory cytokines and adipokines, a study in a mouse model of chemically induced colitis-associated colorectal cancer (CAC) showed that diet-induced obesity increased IL-6 levels, thereby favoring macrophage polarization towards tumor-promoting M2 macrophages. Moreover, M2 macrophages produced CC-chemokine-ligand-20 (CCL-20) that promoted the recruitment of CC-chemokine-receptor-6 (CCR-6)-expressing B and T cells. The recruited B cells promoted further macrophage polarization and the T cells inhibited other immune cells, thereby favoring CAC progression [32]. Survivin is also secreted by adipose tissue and drives protumoral macrophage polarization in colon cancer, colorectal adenocarcinoma and hepatocellular carcinoma. Indeed, adipose-derived stem cells (ASCs) from subjects with obesity release survivin that induces tumor-associated macrophage (TAM) reprogramming coincident with increased survivin expression. Thus, survivin produced by ASCs and macrophages together promotes the malignancy of colon and liver cancer cells [33]. Obesity has also been associated with the secretion of adipokines and cytokines in the liver tumor microenvironment. The proinflammatory adipokines visfatin and resistin are increased in the serum and liver tumors of patients with obesity. Moreover, visfatin and resistin increase cell viability, invasion, lipogenesis, fatty acid synthase (FASN) protein levels, MMP-9 activity, as well as Akt and ERK phosphorylation in liver cancer cells [11]. In a mouse model of hepatocellular carcinoma initiated by diethylnitrosamine administration and promoted by dietary or genetic obesity, PI3Kγ ablation reduced tumor growth, insulinemia, and steatosis [34], suggesting a preponderant role for PI3K/Akt signaling in obesity-induced cancer.

## 3. Paracrine Communication between Adipocytes, Macrophages and Cancer Cells in the Tumor Microenvironment

Tumors are now recognized as containing many different cell populations beyond cancer cells, including fibroblasts, immune cells (macrophages, lymphocytes, neutrophils), endothelial cells and adipocytes, among others [51,52]. Moreover, it is increasingly evident that these additional populations of tumor stromal cells promote extracellular matrix remodeling, cell migration, angiogenesis, invasion, metastasis, and drug resistance by producing a variety of signaling molecules that include extracellular matrix constituents, growth factors, metabolites, chemokines and cytokines [53]. Therefore, to determine what drives tumor development, it is necessary to understand how the complex communication between tumor cells and the surrounding stroma components conspires to produce “factors” that augment inflammation in the tumor microenvironment (TME), thereby favoring tumor progression and metastasis. Note that the mechanisms described in the following paragraphs are summarized in Table 2.

Adipocytes play a fundamental role in the TME of multiple types of cancer, especially in those with significant adipocyte content in the stromal microenvironment. Available evidence indicates that dynamic communication between tumor cells and adipocytes leads to phenotypic and functional changes in both cell types that can drive tumor progression [64,66,88]. This section will consider some of the crosstalk mechanisms between adipocytes and cancer cells in the TME. 

### 3.1. Effects of Adipocytes on Cancer Cells

As mentioned above, communication between cancer cells and adipocytes is reciprocal. Adipocytes have been shown to produce factors involved in matrix remodeling, metabolic reprogramming, EMT, angiogenesis, invasion and the survival of cancer cells as well as drug resistance [56,68,76,79,89]. 

Cancer-associated adipocytes (CAAs) produce elevated insulin-like growth factor binding protein 2 (IGFBP-2) levels in the tumor microenvironment, which upregulates MMP-2 and then promotes migration, invasion, and metastasis in breast cancer [58]. Likewise, CAAs cocultured with breast cancer cells have been shown to increase their expression of MMP-11 as well as proinflammatory cytokines (IL-6 and IL-1β) and thereby promote tumor cell invasion and metastasis [64]. Other investigators demonstrated that cancer cells cultured with in vitro differentiated adipocytes or proinflammatory cytokines (IL-6, IL-8, CXCL10, CCL2 or CCL5) activate Src to upregulate Sox2 and induce miR-302b, generating feed-forward loops that contribute to mammary cancer stem cell self-renewal and drive metastatic tumor progression [61]. Moreover, the interaction between CAAs and breast cancer cells involves a positive feedback loop between the cytokine leukemia inhibitory factor (LIF) and CXCL subfamily chemokines, which promotes breast cancer invasion and metastasis [55]. Furthermore, the expression of factors involved in cancer development, such as MMP-9 [17] and interferon γ-inducible protein 30 [90], show the highest upregulation in CLS+ as compared to CLS- human adipose tissue explants [7]. Moreover, studies in high-fat diet-fed mice have shown that secreted factors from their CLS+ adipose tissue, which is rich in proinflammatory cytokines, induce ROS production, changes in cell morphology, loss of E-cadherin, proliferation and neoplastic transformation in the epidermal JB6 P+ cell line [75].

The evidence also suggests that adipocytes mediate proangiogenic events in several tumors. For instance, IL-1β secreted by macrophages induced ANGPTL4 expression in primary mouse adipocytes through activation of NF-κB and MAPKs, which is further enhanced by hypoxia. Consequently, adipocyte-derived ANGPTL4 promotes obesity-driven breast cancer progression and angiogenesis [39,40]. Additionally, CAAs secrete high levels of IL-8, which increases angiogenesis at the primary tumor site, induces a protumoral neutrophil phenotype, modifies the expression of cell-adhesion molecules in tumor cells and favors early dissemination of breast cancer [59]. Furthermore, Al-Khalaf et al. reported that CAAs secreted higher levels of IL-8, which was crucial for enhancing the proangiogenic effects of breast adipocytes [60]. 

Interestingly, it has been suggested that adipocytes induce metabolic reprogramming in tumor cells. Peritoneum-derived adipocytes induce lipid droplet accumulation and fatty acid oxidation (FAO) in gastric cancer cells through the upregulation of diacylglycerol acyltransferase 2 (DGAT2) in a CCAAT/enhancer-binding protein alpha (C/EBPα)-dependent manner [78]. The lipid-rich environment stimulates nicotinamide adenine dinucleotide phosphate (NADPH) production, promotes resistance to anoikis through ROS-dependent mechanisms, and supports HFD-induced peritoneal dissemination and lung metastasis in vivo [78]. In an adipose triglyceride lipase/hormone-sensitive lipase (ATGL/HSL-dependent manner, adipocytes release fatty acids to boost fatty acid metabolism in breast cancer cells through the increased expression of carnitine palmitoyl transferase I (CPT1A) together with proteins of the electron transport chain, resulting in increased proliferation, migration and invasion of breast cancer cells [65,66]. Mammary adipocytes stimulate metabolic remodeling in cancer cells. Indeed, fatty acid metabolism in cancer cells is uncoupled from ATP production, leading to the promotion of the Warburg effect and increased glycolysis, thereby promoting the proliferation, migration and invasion of cancer cells [66]. Consistently, CPT1A is upregulated in colon cancer cells following coculture with mature human adipocytes or exposure to fatty acids. Elevated CPT1A allows cancer cells to produce increasing levels of Ac-CoA through the mitochondrial FAO to promote activation of the β-catenin/Wnt signaling and cancer stem cell function [80]. Furthermore, human omental adipocytes may act as a source of oleic acid for gastric cancer cells in omental gastric cancer metastases. Oleic acid enhances the invasiveness of gastric cancer cells, as well as the expression of MMP-2, by activating the PI3K-Akt signaling pathway in a PTEN-independent manner [77]. Primary mammary gland-derived adipocytes promote the tumorigenicity of cancer cells that express monocarboxylate transporter 2 (MCT2) via β-hydroxybutyrate. In detail, β-hydroxybutyrate secreted from adipocytes increases histone H3K9 acetylation and induces IL-1β and lipocalin 2 (LCN2) expression to enhance tumorigenicity in MCT2-expressing breast cancer cells, which in turn is linked to poor prognosis in breast cancer patients [54].

The fatty acid receptor CD36 is overexpressed in many metastatic tumors, such as oral squamous cell carcinomas (OSCC), melanoma and breast cancers. In OSCC cell lines or patient-derived cells with low metastatic potential, high levels of CD36 significantly increased lymph node metastasis. Furthermore, metastatic potential depends on the increased expression of FAO-related genes and is boosted by an HFD in a CD36-dependent manner. Consistent with these data, CD36 expression strongly correlates with poor prognosis in lung, bladder and breast cancer patients [82]. Later, the same group observed that ovarian cancer cells cocultured with primary human omental adipocytes expressed high levels of CD36, thereby facilitating exogenous fatty acid uptake and ovarian cancer metastasis. Conversely, CD36 inhibition reduced microenvironment-derived fatty acid uptake in ovarian cancer cells, decreasing adipocyte-stimulated invasion and migration in vitro, as well as tumor growth in vivo [69]. These observations highlight the crucial role of lipid metabolism in tumor progression and metastasis. 

Additionally, adipocytes can induce EMT in several types of cancer. In breast cancer cells, mature adipocytes induce the EMT phenotype and promote cancer cell migration, invasion and proliferation, which could be associated with the upregulation of MMP-9 and twist-related protein 1 (TWIST1) [57]. Melanoma cells cocultured with differentiated 3T3-L1 adipocyte cells increased their invasive ability by promoting the expression of EMT genes (SNAI1, MMP-9, TWIST, and vimentin) and decreased the expression of genes, such as E-cadherin and Kiss1 [73]. Similarly, the conditioned medium of CAAs has been shown to improve migration/invasion and chemoresistance, as well as to promote EMT in pancreatic cancer cells by the upregulation of serum amyloid A1 (SAA1) [81], which has been associated with poor prognosis in breast cancer [91]. 

Other studies related to therapy resistance established that the conditioned media from human adipocytes promote resistance of melanoma cells to chemotherapeutic drugs (cisplatin and docetaxel) and therapeutic agents targeting the PI3K/Akt and MEK/ERK pathways [74]. Similarly, another study showed that human adipocytes of both subcutaneous and visceral/omental origin secrete soluble factors that increase resistance to chemotherapeutic drugs in ovarian cancer cells by activating the Akt pathway [70]. Moreover, primary human omental adipocytes induce FABP4 expression in ovarian cancer cells and promote metastasis and Carboplatin resistance in ovarian cancer cells [67]. On the other hand, for breast cancer cells cocultured with in vitro differentiated murine adipocytes, a significant increase in IL-6 expression was observed, which elevated checkpoint kinase 1 (Chk1) phosphorylation and promoted radioresistance in tumor cells [62]. Together, these studies highlight the role of adipocytes in inducing responses in tumor cells that enhance therapy resistance.

Finally, recent studies showed that murine and human adipocytes release EVs that deliver enzymes and substrates (fatty acids) involved in FAO to melanoma cells, which subsequently improves mitochondrial metabolism and remodels the mitochondrial network to support melanoma migration and invasion [71,72]. Another study reported that EVs from CAAs delivered microRNA-21 (miR21) to ovarian cancer cells, where it suppresses apoptosis and induces chemoresistance to paclitaxel through the downregulation of miR21’s direct target apoptotic peptidase activator factor 1 (APAF1) [68]. Moreover, it has been reported that in vitro differentiated adipocytes as well as primary mammary adipocytes from patients with normal weight or with obesity confer a multidrug resistance phenotype to breast cancer cells by increasing the nuclear efflux of doxorubicin through a major vault protein (MVP)-dependent process as well as by expulsion from breast cancer cells via EVs [63]. 

### 3.2. Effects of Tumor-Associated Macrophages on Cancer Cells

As mentioned above, the communication between cancer cells and TAMs is bidirectional. In the TME, TAMs typically promote cancer cell proliferation, angiogenesis, metastasis, and other processes through various anti-inflammatory mechanisms [92]. 

TAMs are known to be strongly involved in tumor angiogenesis. For instance, it has been reported that the presence of M2-polarized TAMs and Tie2-expressing monocytes is associated with angiogenesis, as well as decreased overall and recurrence-free survival in human PDAC patients [86]. In pancreatic islet cancers, breast tumors and lung metastases, interleukin-4 (IL-4) induces cathepsin protease activity in macrophages. Consequently, cathepsins B and S supplied by TAMs promote tumor growth, angiogenesis and invasion in vivo, as well as markedly increase the invasiveness of cancer cells in vitro [85]. Others reported that the development of human breast cancer is strongly associated with overexpression of the Wnt family ligand Wnt7B in the tumor stroma and that isolated human breast carcinoma TAMs express Wnt7B. Furthermore, in mice, Wnt7b was shown to play a crucial role in the malignant progression of luminal breast cancer by promoting angiogenesis, tumor growth, progression, invasion and metastasis. Additionally, the authors attributed the failure to induce the angiogenic switch to the reduced VEGF-A mRNA and protein levels in vascular endothelial cells [83]. 

M2-like TAMs reportedly promote glycolysis and hypoxia in cancer cells, leading to changes in cellular metabolism that are necessary for tumor initiation and progression. In non-small cell lung cancer, TAMs secrete TNFα to promote glycolysis in tumor cells and activate both AMPK and peroxisome proliferator-activated coactivator gamma 1-alpha, which together augment tumor hypoxia by promoting mitochondrial oxygen consumption and increasing mitochondrial membrane potential [87]. Furthermore, in this same study, TAMs were found to significantly reduce T cell infiltration by upregulating programmed death ligand 1 (PD-L1) expression in tumors. Therefore, the immune mechanisms that are associated with PD-L1 expression and controlled by TAMs can be considered critical for tumor immune escape [93]. Importantly, lactate efflux plays a critical role in tumor–stroma communication. Cancer cell-derived lactate increases the secretion of CCL5 through Notch signaling in TAMs, and CCL5 in turn induces EMT and aerobic glycolysis in breast cancer cells. In addition, TGF-β signaling regulates the expression of the CCL5-CCR5 axis components, which induce aerobic glycolysis in cancer cells by activating AMPK signaling. These observations point towards a pivotal role for the CCL5-CCR5 axis in metabolic communication between cancer cells and macrophages [84]. 

Finally, differences between TAMs from primary and metastatic cancers have been reported. In the primary tumor, TNFα induces EMT and the expression of IL12Rβ, a subunit of the IL-35 receptor, to promote tumor cell migration and invasion. Instead, at metastatic sites, TAMs secrete interleukin-35 (IL-35) to activate the JAK2–STAT6–GATA3 signaling axis in cancer cells, which reverses EMT to facilitate tissue colonization by cancer cells [94]. Additionally, Morrissey et al. reported that tumor-derived EVs polarize premetastatic niche macrophages towards an immunosuppressive phenotype through NF-kB-dependent glycolytic reprogramming to favor tumor cell metastasis. These prometastatic macrophages are characterized by increased de novo synthesis of PD-L1, increased glucose uptake, and GLUT-1 expression as well as the increased conversion of pyruvate to lactate, which subsequently augments further PD-L1 expression [95].

## 4. Paracrine Communication between Cancer Cells, Adipocytes and Macrophages in the Tumor Microenvironment

Macrophages in the tumor microenvironment, termed TAMs, represent the largest immune cell population in most human solid tumors [96,97,98]. TAMs are implicated in tumor progression through various mechanisms, such as promoting tumor growth, angiogenesis, invasion and metastasis as well as mediating resistance to drug therapy and suppressing antitumor immune responses [99,100]. Cancer cells and macrophages communicate with one another to determine the local organization of the TME. Therefore, in this section, we will discuss studies that explain how cancer cells recruit macrophages and change their phenotype to TAMs and how TAMs then modulate the characteristics of cancer cells to favor tumor progression. Note that the mechanisms described in the following paragraphs are summarized in Table 3.

### 4.1. Effects of Cancer Cells on Adipocytes in the Tumor Microenvironment

In this section, we will focus on recent studies that explain how normal adipocytes are “transformed’ into CAAs by tumor cells and how adipocytes regulate cancer cell behavior in the TME, thereby supporting tumor progression. Several reports suggest that invasive tumor cells modify the phenotype of adipocytes by reprogramming them to become CAAs, which in turn stimulate the aggressive behavior of cancer cells [64,125]. CAAs are located primarily around the invasive front of tumors, that is, adjacent to cancer cells. Morphologically, CAAs are smaller in size than normal adipocytes and have irregular shapes and smaller lipid droplets [64,126]. Thus, CAAs exhibit a modified phenotype characterized by the loss of lipid content, a decrease in mature adipocyte markers, as well as changes in the expression of adipokines, cytokines, and inflammatory factors, such as leptin, matrix metalloproteinase-11 (MMP-11), CCL2, chemokine ligand 5 (CCL5), IL-6 and IL-1β [64,127]. Compared to normal mature adipocytes, CAAs possess biological characteristics that promote metabolic reprogramming of cancer cells, extracellular matrix remodeling, tumor growth, progression and metastasis. Importantly, the acquisition of many of these characteristics is often also linked to changes in the adipocyte secretome [64,66,125]. Moreover, Zoico et al. demonstrated that pancreatic tumors induce the dedifferentiation of adipocytes into fibroblast-like cells through a Wnt5a-dependent signaling pathway. Specifically, the coculture of pancreatic cancer cells and adipocytes induces the JAK/STAT3 pathway, leading to increased Wnt5a expression and adipocyte reprogramming [107]. Likewise, breast cancer cells secrete Wnt3a, which activates the Wnt/β-catenin pathway and induces the dedifferentiation of mouse and human adipocytes, which then acquire a CAA-like phenotype [103]. 

Wei et al. proposed that the interaction between CAAs and breast cancer cells promotes the reorganization of adipocyte-derived collagen in a procollagen-lysine 2-oxoglutarate 5-dioxygenase 2 (PLOD2)-dependent manner, facilitating breast cancer migration along aligned and clustered collagen fibers. Mechanistically, PAI-1 is secreted by breast cancer cells and promotes the expression of PLOD2 in adipocytes by activating the PI3K/AKT-FOXP1 axis. Consequently, PLOD2 activation induces the linear organization of collagen derived from CAAs, which favors tumor cell metastasis [102]. 

The tumor microenvironment may alter adipose lipid metabolism. Dirat et al. demonstrated that mature adipocytes cocultured with breast cancer cells exhibited an altered phenotype associated with a reduced lipid content and a decrease in adipocyte markers together with an overexpression of MMP-11 and proinflammatory cytokines (IL-6 and IL-1β) [64]. Additionally, breast cancer cells have been reported to drive lipolysis in adipocytes by stimulating the ATGL/HSL pathway [65], thus elevating local free fatty acids. Similarly, in the presence of ovarian cancer cells, adipocytes release significantly more free fatty acids than adipocytes growing alone, thereby increasing the availability of lipids in the tumor microenvironment. As a result, adipocytes provide fatty acids to cancer cells and drive β-oxidation, suggesting that they are converted into CAAs to provide metabolites for cancer cells. Moreover, hypoxic cancer cell-conditioned media potentiate the metabolic shifts in adipocytes by increasing lipolysis in a HIF-1α dependent manner. Thereby, adipocytes transfer lipids to breast and ovarian cancer cells and consequently increase cancer cell proliferation [128]. In addition, adipocytes cocultured with breast cancer cells increase the expression of HIF-1α and TGF-β. Cancer cells cocultured with adipocytes increase the levels of EMT-inducing transcription factors FOXC2 and TWIST1 in a HIF-1α adipocyte expression-dependent manner. Taken together, these results suggest that adipocytes under hypoxic conditions promote cancer occurrence, migration, invasion and metastasis [129]. Additionally, upregulation of fatty acid-binding protein 4 (FABP4, also known as aP2) was observed in omental metastases compared to primary ovarian tumors. This suggests that FABP4 could be key in the ovarian cancer cell–adipocyte interaction, resulting in elevated lipid availability to favor metastasis [106]. In patients with colorectal cancer, a significant reduction in gene expression and activity levels of lipoprotein lipase (LPL) and FASN was detected in adipose tissue adjacent to the tumor lesion compared to tissue distant from the neoplasm [108]. Taken together, these results highlight the importance of the tumor microenvironment in adipose tissue lipid metabolism, as they provide evidence for tumor-induced deterioration in the lipid storage capacity of adipose tissue in patients with colorectal cancer. 

In the last decades, the phenomenon of white adipose tissue browning has gained increasing interest in the obesity field. In this process, white adipocytes acquire a thermogenic phenotype in response to stimuli, such as cold or beta-adrenergic agonists [130]. A few studies support the idea that adipocytes adjacent to cancer cells display higher browning activity, which may contribute to tumorigenesis. Specifically, it has been demonstrated that local activation of tumor-adjacent thermogenic adipocytes could promote cancer cell proliferation and invasion by providing fuel sources [131]. Master et al. demonstrated that the mammary gland contains brown adipose tissue, which is potentially implicated in adaptive thermogenesis [132]. Other studies have shown that the expression of genes encoding for markers of fat browning, such as uncoupling protein 1 (UCP1), PR domain-containing 16 (PRDM16), cell death-inducing DFFA-like effector protein A (CIDEA), cytochrome c oxidase subunit VIIa polypeptide 1 (COX7A1), peroxisome proliferator-activated receptor gamma coactivator 1α, transmembrane protein 26 (TMEM26), and T-box 1 (TBX1), were upregulated in adipose tissue adjacent to breast tumors. Furthermore, the browning activity of adipocytes adjacent to breast cancer tissues was higher compared to those adjacent to benign breast lesions [104]. More recently, tumor cells have been shown to induce beige/brown phenotype and remodel metabolism in resident adipocytes by EVs with elevated miRNA-144 and miRNA-126 content. miRNA-144 promotes the beige/brown phenotype in adipocytes by downregulating the MAP3K8/ERK1/2/PPARγ axis, while miRNA-126 remodels metabolism by disrupting IRS/Glut-4 signaling, activating the AMP-activated protein kinase (AMPK)/autophagy pathway and stabilizing HIF1α expression [101]. Likewise, miR-155 derived from breast cancer cell exosomes promotes browning and metabolic remodeling of adipocytes through the downregulation of PPARγ, which triggers cancer-associated cachexia and facilitates tumor progression [105].

### 4.2. Effects of Cancer Cells on Macrophages in the Tumor Microenvironment

TAMs originate from two primary sources: (a) tissue-resident macrophages that develop from embryonic precursors (fetal yolk sack or fetal liver progenitors) and (b) macrophages derived from circulating monocytes that originate from bone marrow hemopoietic cell progenitors [133]. The TME affects the programming of both resident and infiltrating macrophages such that they acquire functionally distinct phenotypes [134,135]. As mentioned in previous sections, two well-established polarized phenotypes are the classically activated (M1) and the alternatively activated (M2) macrophages [136].

The M1-like polarized macrophages are activated by Th1 cytokines, such as interferon-γ (IFN-γ), or by exposure to lipopolysaccharides (LPS). Classically activated macrophages are responsible for generating a proinflammatory response directed against pathogens by incrementing phagocytic activity combined with the secretion of proinflammatory cytokines (IL-6, interleukin-12 (IL-12), interleukin-23 (IL-23), TNF-α) [137], as well as chemokines (CXCL1–3, CXCL-5, CXCL8–10) [138]. Moreover, they are characterized by a high antigen presentation capacity and produce proinflammatory mediators, such as nitric oxide and ROS [139,140]. After recognition by M1 macrophages, tumor cells can be killed through several mechanisms, including contact-dependent phagocytosis and cytotoxicity [141]. In contrast, M2 macrophages are activated by cytokines, such as IL-4, IL-13, glucocorticoids and immunoglobulin complexes that are anti-inflammatory and regulate tissue remodeling, angiogenesis, wound healing, parasite clearance and immunosuppression [100,142]. The transition of macrophages to the M2 phenotype facilitates tumor development, specifically through the production of anti-inflammatory cytokines (IL-10, arginase-1, TGF-β), as well as proangiogenic factors (CCL2, placental growth factor (PGF) and VEGF-A), which together favor tumor growth and metastasis [92,143,144]. 

Various studies have demonstrated that TAMs in the TME develop tumor-favoring activities similar to those of M2 macrophages [100], including promoting angiogenesis and matrix remodeling, as well as suppressing adaptive immunity. Moreover, they facilitate tumor progression through the secretion of several pro-angiogenic factors, extracellular matrix remodeling molecules, and anti-inflammatory cytokines [145,146]. Notably, the tumor promoting role of TAMs in cancer is supported by clinical studies that observed a positive correlation between the extent of macrophage presence in tumors and poor patient prognosis [147,148]. 

Several microenvironment signals derived from tumor and stromal cells directly affect monocyte recruitment and macrophage polarization. For instance, hypoxia can be a crucial driver of macrophage recruitment and the tumor-supporting transformation of TAMs [149]. In this context, it has been reported that hypoxia increases ZEB1 expression in cervical cancer cells, which directly promotes CCL8 production and induces macrophage infiltration, mainly into hypoxic areas, via the CCR2–NF-κB pathway. On the other hand, CCL8-mediated TAM infiltration contributes to hypoxic ZEB1-related cancer progression and poor prognosis in cervical cancer [114]. Similarly, it has been shown that hypoxic cervical cancer cells instruct the recruited macrophages to transform into M2-like TAMs by upregulating Neuropilin-1 (Nrp-1) in hypoxic regions. The presence of Nrp-1 and M2-like TAMs correlated with an increase in the malignant properties of cervical cancer cells, such as lymph node metastasis and poor tumor differentiation [113]. In breast cancer, hypoxic cancer cells reportedly attract macrophages and induce their M2 polarization by secreting eotaxin and oncostatin M [110]. Damage-associated molecular pattern (DAMP) and high-mobility group box 1 (HMGB1) proteins are also associated with hypoxia-induced macrophage polarization. Huber et al. reported that HMGB1 is released by melanomas in hypoxia and promotes the M2-like TAM phenotype, as well as IL-10 accumulation within a tumor [121]. In addition, 5-lipoxygenase (5-LOX), a member of the lipoxygenase gene family, is a crucial enzyme which converts arachidonic acid to 5-hydroxyeicosatetraenoic acid (5-HETE) and leukotrienes, lipid mediators with essential roles in tumor-associated inflammation [150]. Wen et al. found that the expression of 5-LOX strongly correlated with the density of TAMs in hypoxic areas of human ovarian tumors and provided in vitro evidence that increased 5-LOX metabolites from hypoxic ovarian cancer cells promote TAM infiltration through the upregulation of MMP-7 [112]. Finally, in hypoxic tumor microenvironments, various chemoattractants, such as endothelin-2 [151], VEGF-A [152], and endothelial-monocyte activating polypeptide (EMAPII) [153], also play a potential role in regulating macrophage recruitment and differentiation into TAMs [100,154].

Alterations of metabolic pathways in the tumor microenvironment also regulate macrophage polarization and function. During anaerobic glycolysis, tumor cells secrete lactate, thereby favoring macrophage differentiation to the M2 phenotype, which in turn promotes tumor growth by liberating VEGF-A and arginase-1 [120]. Interestingly, M2 polarization of melanoma-associated TAMs seems to be promoted by a mechanism involving a G-protein-coupled receptor that senses TME acidification induced by enhanced cancer cell glycolysis [119]. Moreover, α-ketoglutarate (αKG) produced via glutaminolysis in bone marrow-derived macrophages (BMDMs) is an anti-inflammatory metabolite that promotes M2 activation and controls metabolic reprogramming of M2 macrophages through jumonji domain-containing protein 3 (Jmjd3)-dependent mechanisms. Furthermore, αKG limits M1 polarization by reducing NF-κB pathway activity via αKG–prolyl hydroxylase (PHD)-dependent control of IKKβ activity [155]. 

In pancreatic ductal adenocarcinoma (PDAC), G12D is the most frequent KRAS mutation (referred to as KRAS^G12D^) and the presence of this oncogene correlates with distinctive features of the TME [156]. Notably, autophagy-dependent ferroptosis favors the EV-mediated release of KRAS^G12D^ protein from PDAC cells during oxidative stress. Upon EV uptake by macrophages via the receptor for advanced glycation end products (RAGE), KRAS^G12D^ induces M2-like TAM polarization via STAT3-dependent fatty acid oxidation. Importantly, the abundance of KRAS^G12D^ in TAMs is associated with poor prognosis of PDAC patients [115]. 

Chemokines, cytokines and products of the complement cascade are significant determinants of macrophage recruitment and accumulation in tumors [157,158]. For instance, elevated levels of tumor-derived chemotactic protein CCL2 have been correlated with high numbers of TAMs and poor patient prognosis in various cancer types [118,123,124]. Moreover, NADPH oxidase 4 (NOX4) in tumors reportedly mediates macrophage chemotaxis and M2 polarization in lung cancer by stimulating the expression of various cytokines, including CCL7, IL-8, CSF-1 and VEGF-C via ROS/PI3K/AKT signaling. Likewise, NOX4-educated M2 macrophages exhibit elevated JNK activity and release heparin-binding EGF (HB-EGF) related growth factor, thus contributing to non-small cell lung cancer growth [122]. Another study revealed that the IL-6 receptor gp130 is present in breast cancer cell-derived EVs and stimulates STAT3 signaling in BMDMs. The survival of BMDMs increases in response to EV exposure, also upregulating the expression of protumoral cytokines, thereby converting them into potentially cancer-promoting macrophages [109].

As previously anticipated, several studies suggest that EVs might participate in the communication between cancer cells and macrophages. For instance, several miRNAs (miR-25-3p, miR-130b-3p, miR-425-5p) that are upregulated in colorectal cancer cells by activating the CXCL12/CXCR4 axis, are transferred to macrophages via EVs and induce their polarization. Specifically, exosomal miRNAs induce M2 polarization by activating the PI3K/AKT signaling pathway and inhibiting PTEN. In turn, M2-polarized macrophages promote liver metastasis of colorectal cancer by enhancing EMT and secreting VEGF [117]. Similarly, epithelial ovarian cancer-derived EVs contain high levels of miR-222-3p that can polarize macrophages to M2-like TAMs and thereby favor tumor progression by inducing SOCS3/STAT3 signaling [111]. Additionally, miR-145 secreted from colorectal cancer cells via EVs is taken up by macrophages where it promotes polarization to the M2-like phenotype through the downregulation of histone deacetylase 11 (HDAC11). Therefore, TAMs polarized by miR-145 contained in EVs contribute to the modulation of the tumor microenvironment and tumor progression [116].

## 5. Antioxidants as Potential Therapeutic Tools Targeting Adipocyte–Macrophage Crosstalk in Cancer 

### 5.1. Effects on Obesity and Cancer

ROS participate in many physiological and pathological processes. For example, oxidative stress modulates the differentiation of adipocytes in physiological conditions and obesity. With this in mind, one might anticipate that antioxidants could be employed for the development of therapeutic strategies to preclude obesity-related oxidative stress (reviewed in [159]). 

The main source of ROS are the mitochondria due to the leakage of electrons from the respiratory chain. In addition, ROS are produced by the endoplasmic reticulum, lysosomes, peroxisomes and cytosolic enzymes [159]. ROS regulate signaling pathways, gene expression and cell death in normal and cancer cells. In normal cells, a balance exists between ROS formation and antioxidant activity that maintains low ROS levels, thereby limiting cell proliferation and survival. Cancer cells increase ROS generation and also antioxidant capacity, which allows them to adapt to elevated ROS levels and pro-tumorigenic signaling without suffering cytotoxic effects. Through the activation of MAPK/ERK and PI3K/Akt signaling pathways, ROS enhance cell proliferation and cell survival. In addition, oxidative stress enhances HIF-1α expression and allows cancer cell adaptation. Furthermore, ROS induce drug resistance by activating PI3K/Akt and HIF-1α. Exacerbated increments in ROS levels to toxic concentrations will also promote cancer cell death (reviewed in [160]). For this reason, increases in ROS and their reduction using antioxidants can be associated with both beneficial and detrimental effects in cancer cells (reviewed in [161]). For instance, some chemotherapies increase ROS to levels that promote oxidative stress-induced tumor cell death while others suppress ROS levels to reduce cancer cell proliferation (reviewed in [160]). 

In previous sections, we have summarized information linking obesity-induced inflammation to cancer development and progression. Indeed, obesity promotes cancer through mechanisms involving ROS and is associated with the presence of adipokines, VEGF, TNFα and IL-6 (reviewed in [162]). For that reason, the coming sections will discuss how antioxidants may protect against adverse obesity-induced effects both at the systemic (Section 5.2) and local levels (Section 5.3).

### 5.2. Systemic Effects

As we previously described, an intricate connection exists between obesity, inflammation and cancer, whereby several systemic mediators and pathways are involved, including but not limited to ROS, cytokines, adipokines, Akt, STAT3 and several metabolites. Table 4 summarizes antioxidants known to prevent cancer associated with obesity and inflammation by controlling these systemic mechanisms.

#### Plant-Derived Antioxidants

Curcumin, a polyphenol with antioxidant and anti-inflammatory properties found primarily in turmeric, has been ascribed interesting beneficial systemic effects. For instance, curcumin combined with the anti-inflammatory drug salsalate reportedly prevents the development of colon cancer in mice fed a HFD and exposed to the colon-specific carcinogen azoxymethane. The combination of curcumin/salsalate elicited such effects by reducing proinflammatory cytokine levels and diminishing activation of the PI3K/Akt/mTOR/NF-κB/Wnt pathway [163,164]. Importantly, curcumin alone has antitumor effects too. For example, curcumin prevents obesity-related colorectal cancer by attenuating chronic inflammation and improving adipokine imbalance, reducing cyclooxygenase-2 (COX-2), AMPK, NF-κB, TNF*α*, IL-6 and leptin levels, while increasing adiponectin levels [165]. In addition, curcumin prevents accelerated polyp development associated with a HFD by enhancing apoptosis, reducing oxidation-induced DNA damage, and increasing the efficiency of DNA repair [166]. 

Similar to curcumin, epigallocatechin gallate (EGCG) is an antioxidant that seems to be effective in reducing obesity and tumor growth. Specifically, EGCG has been shown to suppress azoxymethane-induced premalignant colon lesions in obese mice and these beneficial effects of EGCG were associated with reductions in IGF-IR, p-GSK-3β, β-catenin, COX2 and cyclin D1 protein levels as well as IGF-I, insulin, triglyceride, cholesterol and leptin serum levels [167]. Silibinin, a polyphenolic flavonoid found in silymarin extracted from milk thistle, is another antioxidant that reduces the protumoral properties associated with obesity. In fact, silibinin prevents lipogenesis and obesity-induced tumor progression. Moreover, in hepatocellular carcinoma HepG2 cells, silibinin reduces invasion by decreasing ROS, MMP-9, FASN, IL-6, IL-1β and Erk phosphorylation [168].

Some flavonoids have both antioxidant and antitumoral effects. For instance, obese ovariectomized mice on a HFD supplemented with naringenin have less adipose mass, reduced adipocyte size, as well as inflammatory cytokine levels, in both mammary and perigonadal adipose tissues than their counterparts without supplementation. Moreover, naringenin delays the growth of tumors at the early stages of breast cancer development [169]. Taxifolin is another flavonoid that can prevent obesity-induced breast cancer [170]. In fact, a study in African American women with obesity showed that adipose tissue secretes hepatocyte growth factor (HGF) with an endocrine effect on breast cancer cells. Specifically, HGF activates c-Met, thereby increasing SOS1-Grb2-CCL2 signaling. CCL2, in turn, induces macrophage polarization to favor the development of an anti-inflammatory microenvironment. Likewise, taxifolin inhibits SOS1 signaling by blocking the interaction between SOS1 and Grb2. Thus, taxifolin can prevent the systemic effects of obesity in cancer cells by reducing signaling due to adipose-secreted HGF. In addition, taxifolin prevents the local effects of obesity by inhibiting SOS1 signaling, thereby reducing macrophage polarization in the tumor microenvironment (see Table 5, next section [170]).

Resveratrol is another antioxidant that reduces the endocrine effects of obese adipose tissue on breast cancer cells. More specifically, subcutaneous adipose tissue from obese rats promotes cell cycle entry in MCF7 breast cancer cells. Resveratrol supplementation can prevent this by increasing the adiponectin/leptin ratio as well as promoting AMPK and inhibiting Akt signaling [172]. In addition, resveratrol inhibits obesity-associated adipose tissue dysfunction and tumor growth. In a mouse model of diet-induced obesity, abnormal adipose expansion was associated with a deregulated COX-2/PPARγ ratio, increased levels of proinflammatory markers, macrophage infiltration and tumor growth. Supplementation with resveratrol attenuates the effects of diet-induced obesity by inhibiting adipocyte hypertrophy and dysregulation and also prevents breast cancer development [171]. Of note, resveratrol not only has systemic but also local effects and modulates adipocyte function in the tumor microenvironment, as summarized in Table 5.

### 5.3. Local Effects

In the previous section, we summarized some examples of the impact obesity has on the tumor microenvironment, underscoring the relevance of systemic and local events in the development of cancer. Within the tumor microenvironment, cancer cell interactions with adipocytes and macrophages promote cancer progression through a variety of mechanisms involving oxidative stress, IL-6, IL-1β, STAT3, ERK and CLS. In the following paragraphs, we summarize some of the available literature evidencing the efficacy of antioxidant treatments in reverting/preventing cancer by remodeling the tumor microenvironment and regulating the mechanisms summarized in Table 5.

As may be anticipated, some antioxidants, such as taxifolin and resveratrol, have systemic and local effects that contribute to preventing obesity-induced inflammation and cancer [170,171]. For instance, taxifolin inhibits the stimulation of cancer cells by HGF secreted by adipocytes and does so by preventing activation of the SOS1-Grb2-CCL2 pathway. In this way, taxifolin reduces CCL2 in cancer cells and modulates macrophage polarization, thereby preventing cancer progression [170]. Another example is resveratrol, which reduces the median area of intratumoral adipocytes and the number of TAMs that increase as a consequence of diet-induced obesity. Thus, resveratrol modifies the tumor microenvironment by preventing the formation of CLS that appear as a consequence of adipocyte dysfunction and macrophage infiltration. In doing so, resveratrol reduces obesity-related cancer development [171]. 

Recently, Stassi and collaborators reviewed novel strategies targeting adipose tissue-released factors such as lipids, glucose, adipocytokines and miRNA [185]. For instance, targeting lipid biosynthesis through FASN inhibition results in effective antitumoral effects [185]. Indeed, silibinin reduces FASN protein expression, thereby decreasing the impact of obesity in liver cancer [168]. In addition, PPARγ regulates lipid and glucose homeostasis, as well as the expression of tumor suppressor genes, such as BRCA1 and PTEN. In this regard, PPARγ antagonists impair cell proliferation and mammary tumor growth [185]. In fact, in silico analysis demonstrates that quercetin and rutin target PPARγ and thereby have potential anti-obesity effects [186]. Targeting MTC1 (a lactate transporter) or lactate dehydrogenase, and thereby controlling glucose metabolism, reduces the occurrence and progression of non-small cell lung cancer [185]. Moreover, resveratrol increases insulin signaling and glucose uptake by adipocytes [187], indicative of its potential therapeutic benefits in treating adipocyte-altered glucose metabolism. Finally, antioxidants play a relevant role in the treatment of obesity. For instance, rosiglitazone combined with manganese tetroxide antioxidant nanoparticles synergistically reduces obesity via locally promoting beige adipogenesis, reducing fat accumulation and increasing antioxidative defense in adipose tissue. The mechanisms implicated involve the inhibition of PPARγ and ROS scavenging [188]. Similarly, the antioxidant arctigenin reduced the weight gain induced by HFDs in obese mice through the inhibition of adipogenesis-related factors, including PPARγ, C/EBPα, fatty acid synthase, adipocyte fatty acid-binding protein and lipoprotein lipase [189].

In colon cancer, nanoparticles containing vitamin E succinate inhibit cancer cell proliferation and dissemination as well as improve the intraperitoneal microenvironment by decreasing the levels of VEGF, IL-10 and the presence of M2-like TAMs [173]. Furthermore, the polyphenolic isoflavone found in soybeans, genistein, regulates the tumor microenvironment and exhibits an anticancer effect in oxidative stress-induced experimental colon carcinogenesis [174]. In addition, berberine is another antioxidant that inhibits the development of colitis-associated colorectal cancer by interfering with TNFα and IL-6-induced inflammatory responses in colonic macrophages, thereby inhibiting EGFR/ERK signaling in tumor cells [175]. Moreover, berberine downregulates TNFα and other inflammatory and procarcinogenic markers (IL-1β, Ccl1, Cxcl9, NF-κB, COX-2, JNK/STAT3, β-catenin, c-Myc and CylinD1), as well as modulates gut microbiota. In fact, berberine increases the abundance of beneficial bacteria, such as Roseburia, Eubacterium, Ruminococcaceae and Firmicutes, while reducing detrimental Odoribacter, Muribaculum, Mucispirillum and Parasutterella. These changes aid in preventing tumor development by decreasing the release of proinflammatory and carcinogenic factors [176,177]. Berberine, likewise, reverts breast cancer by attenuating the inflammatory response. Specifically, berberine reduces the expression of TNFα and IL-6 as well as inhibits c-Jun, c-Fos and NF-κB signaling in breast cancer cells [178]. 

EGCG is another antioxidant that is suggested to prevent breast cancer. First, EGCG was reported to inhibit adipocyte/cancer cell STAT3-mediated oncogenic paracrine control, thereby preventing adipogenesis and the obesogenic environment that favors breast cancer development [179]. Furthermore, EGCG was found to modulate not only adipogenesis but also inflammation. Breast cancer cells induce the expression of cytokines (CCL2, CCL5, IL-1β, and IL-6) and immunomodulators (COX2, HIF-1α, VEGFα, and PD-L1) by cancer-associated adipocytes. EGCG prevents this inflammatory response due to the cancer cell–adipocyte connection and also modulates Snail, Smad2 and NF-κB [180]. Moreover, EGCG has been successfully employed in the release of nanochemical drug combinations. For instance, the EGCG/taxane combination promotes the internalization of the nanoparticles into prostate cancer cells through CD44 and the inhibition of cell growth [181]. 

Among other antioxidants, quercetin exerts beneficial effects in a xenograft model of liver cancer via the remodeling of the tumor microenvironment in a manner that improves tumor permeation by quercetin/doxorubicin-loaded nanoparticles [182]. Additionally, extracellular microparticles encapsulated with the organic compound diallyl trisulfide found in garlic prevent the prometastatic, inflammatory microenvironment in lung metastatic niches. These extracellular microparticles are able to fuse with cancer and normal lung cells and inhibit tumor cell migration, as well as reduce inflammation in the tumor microenvironment by decreasing the release of S100A8/A9 and IL-6 [183]. Similarly, the synthetic antioxidant nicaraven prevents the growth of inflamed lung tumors by reducing the recruitment of macrophages and neutrophils. Indeed, Nicaraven reduces IL-2 and MIP-2 levels in serum, as well as CXCL10 and SDF-1 levels in tumors [184].

## 6. Conclusions

Obesity is a complex multifactorial disease that is associated with the development of multiple comorbidities and, as such, it has become of great importance to better understand the molecular underpinnings involved in these processes. In the context of this review, the connection that exists between obesity and the development of several types of cancer is of particular interest (see Figure 1). Specifically, inflammatory changes that adipose tissue undergoes in obesity are linked to multiple effects on cancer cell behavior. These changes can be triggered by systemic “endocrine” signaling from adipose tissue to the tumor or by more intimate “paracrine” mechanisms in the tumor microenvironment. The prevalent macrophages and mechanisms are summarized showing how the presence of tumor-associated adipocytes “convert” these macrophages into a population of cells that very actively promote tumor growth. Importantly, the signaling between these two cell populations is bidirectional and serves to generate an amplification loop that exacerbates inflammation and oxidative stress in the tumor environment. This in turn favors cancer cell progression to an ever more aggressive phenotype and ultimately promotes metastasis, the leading cause of death in cancer patients. Fortunately, antioxidants of vastly different chemical natures have shown some promise in blocking several of these mechanisms, and one may anticipate that gaining greater insight into how precisely such agents function in the aforementioned context could yield key “reagents” to detain tumor development.

## Figures and Tables

**Table 1:** Endocrine communication between adipose tissue and the tumor microenvironment.

Cancer Type	Mechanism	Refs.
Increased cancer-associated mortality	Inflammation and metabolism	[31]
Colitis-associated cancer	IL-6, CCL-20/CCR-6	[32]
Colon cancer, colorectal adenocarcinoma, and hepatocellular carcinoma	Survivin	[33]
Liver cancer	Visfatin and Resistin Akt and ERK, ROS, MMP-9	[11]
Hepatocellular carcinoma	PI3Kγ, insulinemia and steatosis	[34]
Liver and uterine cancer	microRNA, fatty acid and arachidonic acid metabolism, cell growth, and inflammation	[35]
Endometrial Cancer	Leptin, proinflammatory cytokines (IL-6) and oxidative stress	[36]
Breast cancer	Leptin	[37]
Breast cancer	Relationship between adipocytes, immune cells and the tumor	[38]
Breast cancer	NLRC4, IL-1β, VEGF, angiogenesis	[39]
Breast cancer	ANGPTL4, IL-1β, angiogenesis, macrophage tumor infiltration	[40]
Breast cancer	PPARα and FAK-signaling (inflammation and lipid metabolism, such as PPARα, IL-1β, and ANGPTL4)	[41]
Breast cancer	White adipose tissue inflammation, higher serum lipid, glucose, C-reactive protein levels and CLS	[42]
Breast cancer	White adipose tissue inflammation, CLS, insulin, glucose, triglycerides, HDL, leptin, adiponectin, C-reactive protein, and IL-6	[43]
Breast cancer	GM-CSF and MMP-9	[44]
Breast cancer	IL-5 and GM-CSF	[45]
Breast cancer	IL-6, SphK1/S1P/S1PR1	[46]
Lung cancer	Lipid metabolism and oxidative stress	[47]
Prostate Cancer	IL-6, MDSCs, M2/M1 tumor infiltration, pSTAT3	[48]
Prostate Cancer	IL-6, VEGF, CXCL1/2/13	[49]

ANGPTL4: angiopoietin-like 4; CCL-20: CC-chemokine-ligand-20; CCR-6: CC-chemokine-receptor-6; CLS: crown-like structures; CXCL1/2/13: C-X-C motif chemokine ligand 1/2/13; FAK: focal adhesion kinase; GM-CSF: granulocyte-macrophage colony-stimulating factor; IL-1β: interleukin-1β; IL-5: interleukin-5; IL-6: interleukin-6; MDSCs: myeloid-derived suppressor cells; MMP-9: matrix metalloproteinase 9; NLRC4: NOD-like receptor 4; PI3K: phosphoinositide 3-kinase; PPARα: peroxisome proliferator-activated receptor alpha; ROS: reactive oxygen species; S1P: sphingosine-1-phosphate; S1PR1: sphingosine-1-phosphate receptor 1; SphK1: sphingosine kinase 1; STAT3: signal transducer and activator of transcription 3; VEGF: vascular endothelial growth factor.

**Table 2:** Paracrine communication between adipocytes, macrophages, and cancer cells in the tumor microenvironment.

**Signals from Adipocytes to Cancer Cells**			
**Cancer Type**	**Mechanisms**	**Effect on TME**	**Refs.**
Breast cancer	β-hydroxybutyrate increases histone acetylation and upregulates the expression of tumor-promoting genes (IL-1β and LCN2)	Tumor growth and poor prognosis	[54]
Breast cancer	A positive feedback loop between the cytokines LIF and CXCLs	Invasion and metastasis	[55]
Breast cancer	G-CSF/Stat3	EMT, migration, and invasion	[56]
Breast cancer	Upregulation of MMP9, TWIST1 and Vimentin	EMT, migration, and invasion	[57]
Breast cancer	IGFBP-2 and MMP-2	Adipocytes acquire CAA-like phenotype. Migration, invasion, and metastasis.	[58]
Breast cancer	IL-1β/ANGPTL4	Tumor growth and angiogenesis	[40]
Breast cancer	IL-8	Angiogenesis and breast cancer cell dissemination	[59]
Breast cancer	IL-8/STAT3	Angiogenesis and tumorigenesis	[60]
Breast cancer	Cytokines (CCL2, CCL5, IL-6, IL-8, CXCL10) activate Src to upregulate Sox2 and induce miR-302b	Cancer stem cell self-renewal and metastasis	[61]
Breast cancer	IL-6/Chk1 phosphorylation	Radioresistant phenotype	[62]
Breast cancer	Major vault protein	Resistance to Doxorubicin	[63]
Breast cancer	Increase expression of MMP-11, IL-6, and IL-1β	Invasion and metastasis	[64]
Breast cancer	ATGL/HSL-mediated fatty acids released by adipocytes. FAO induction through increased CPT1A in cancer cells.	Proliferation and migration	[65]
Breast cancer	ATGL-dependent lipolysis and uncoupled FAO	Invasion	[66]
Breast cancer	NLRC4 inflammasome/IL-1β/VEGFA	Angiogenesis	[39]
Ovarian cancer	FABP4	Metastasis and Carboplatin resistance	[67]
Ovarian cancer	Exosomal miR21/APAF1	Invasion and Paclitaxel resistance	[68]
Ovarian cancer	CD36/Exogenous fatty acid uptake	Migration, invasion, and metastasis	[69]
Ovarian cancer	Arachidonic acid/Akt pathway	Cisplatin resistance	[70]
Melanoma	Adipocyte-EVs transfer proteins implicated in FAO	Metabolic reprogramming, migration and invasion	[71]
Melanoma	Adipocyte-EVs transfer proteins required for FAO	Migration	[72]
Melanoma	Expression of EMT genes (SNAI1, MMP-9, TWIST and Vimentin)	Invasion	[73]
Melanoma	PI3K/Akt and MEK/ERK pathways	Resistance to chemotherapeutic drugs (Cisplatin and Docetaxel)	[74]
Skin cancer	Adipokine, ROS, E-cadherin, TWIST	Proliferation and tumor growth	[75]
Prostate cancer	CCR3/CCL7 axis	Migration	[76]
Gastric cancer	Oleic acid/PI3K/Akt/MMP2	Increase in lipid uptake and invasion	[77]
Gastric cancer	DGAT2-dependent lipid droplet accumulation and redox homeostasis	Proliferation, migration and invasion	[78]
Colon cancer	Upregulation of autophagy and mitochondrial FAO via AMPK	Migration and EMT	[79]
Colon cancer	CPT1A-dependent FAO promote the acetylation and nuclear translocation of β-catenin	Promote cancer stem cell functions, cell proliferation and tumor growth	[80]
Pancreatic cancer	SAA1	Adipocytes acquire CAA-like phenotype and induce migration, invasion, and EMT in cancer cells	[81]
Oral squamous cell carcinoma, melanoma, breast cancer	CD36/FAO-related genes	Poor prognosis. Lymph node metastasis	[82]
**Signals from Macrophages to Cancer Cells**			
**Cancer Type**	**Mechanisms**	**Effect on TME**	**Refs.**
Breast cancer	WNT7B /VEGFA	Angiogenesis, tumor growth, invasion and metastasis	[83]
Breast cancer	Lactate and CCL5-CCR5 axis	Aerobic glycolysis and EMT	[84]
Pancreatic, breast, and lung cancer	IL-4-inducing cathepsin activity in macrophages	Tumor growth, angiogenesis and invasion	[85]
Pancreatic ductal adenocarcinoma	M2-polarized TAMs, angiopoietin-axis and TIE2-expressing monocytes	Angiogenesis and metastasis	[86]
Lung cancer	AMPK/PGC-1α, TNFα, PD-L1	Aerobic glycolysis, tumor hypoxia and resistance to anticancer therapies	[87]

AMPK: AMP-activated protein kinase; ANGPTL4: angiopoietin-like 4; APAF1: apoptotic peptidase activator factor 1; ATGL: adipose triglyceride lipase; CCL2: CC-chemokine-ligand-2; CCL5: CC-chemokine-ligand-5; CCR3: CC-chemokine-receptor-3; CCR5: CC-chemokine-receptor-5; Chk1: checkpoint kinase 1; CPT1A: carnitine palmitoyl transferase I; CXCLs: C-X-C motif chemokine ligands; DGAT2: diacylglycerol acyltransferase 2; EMT: epithelial-to-mesenchymal transition; EVs: extracellular vesicles; G-CSF: granulocyte colony-stimulating factor; IGFBP-2: insulin-like growth factor binding protein 2; IL-1β: interleukin-1β; IL-6: interleukin-6; IL-8: interleukin-8; LCN2: lipocalin 2; LIF: leukemia inhibitory factor; MMPs: matrix metalloproteinase; NLRC4: NOD-like receptor 4; PD-1: programmed cell death-1; PD-L1: programmed death ligand 1; PGC-1α: peroxisome proliferator-activated coactivator gamma 1-alpha; PPARγ: peroxisome proliferator-activated receptor gamma; SAA1: serum amyloid A1; TNFα: tumor necrosis factor alpha; TWIST1: twist-related protein 1; VEGFA: vascular endothelial growth factor A.

**Table 3:** Paracrine communication between cancer cells on adipocytes and macrophages in the tumor microenvironment.

**Signals from Cancer Cells to Adipocytes**			
**Cancer Type**	**Mechanisms**	**Effect on TME**	**Refs.**
Breast cancer	Lower C/EBPα expression IL-6/CCL2	Adipocytes revert to an immature and proliferative phenotype. Proliferation, migration and EMT	[88]
Breast cancer	IL-6	Adipocytes acquire CAA-like phenotype and promote invasion in cancer cells	[64]
Breast cancer	ATGL-dependent lipolysis and uncoupled FAO	Induce a lipolytic process in adipocytes, which then become CAA. Invasion and metastasis	[66]
Breast cancer	Exosomal miRNA-144 and miRNA-126	Induce beige/brown differentiation in adipocytes. Metabolic reprogramming and tumor progression	[101]
Breast cancer	PAI-1, PLOD2, and PI3K/AKT-FOXP1 axis	Adipocyte-derived collagen reorganization, which leads to invasion and metastasis	[102]
Breast cancer	Wnt/β-catenin pathway	Adipocytes acquire CAA-like phenotype	[103]
Breast cancer	Increases expression of fatty browning marker genes in adipose tissue adjacent to tumors	Browning activity of adipocytes adjacent to cancer tissues	[104]
Breast cancer	Exosomal miR-155/PPARγ	Browning and metabolic remodeling of adipocytes. Cancer-associated cachexia and tumor progression	[105]
Ovarian cancer	HSL and FABP4	Adipocyte lipolysis. Tumor growth and metastasis	[106]
Pancreatic cancer	WNT5a	Adipocytes acquire CAA-like phenotype	[107]
Colorectal cancer	Reduction of LPL and FASN in adipose tissue adjacent to tumors	Impairs lipid storage capacity of adipose tissue	[108]
**Signals from Cancer Cells to Macrophages**			
**Cancer type**	**Mechanisms**	**Effect on TME**	**Refs.**
Breast cancer	Breast cancer-derived EVs /IL-6/gp130/STAT3	Induce TAM polarization	[109]
Breast cancer	Hypoxia/Eotaxin and Oncostatin	Macrophage recruitment, M2 polarization and angiogenesis	[110]
Ovarian cancer	Ovarian cancer-derived exosomes /SOCS3/STAT3 pathway	Activate macrophages to a TAM phenotype. Tumor growth and metastasis.	[111]
Ovarian cancer	5-LOX metabolites enhance the expression of MMP-7 through activation of the p38 pathway	Macrophage infiltration. Metastasis.	[112]
Cervical cancer	Hypoxia/Nrp-1	Macrophage recruitment and M2 polarization	[113]
Cervical cancer	Hypoxia increases ZEB1 expression, which promotes CCL8 production via the CCR2–NF-κB pathway	Macrophage infiltration into hypoxic areas	[114]
Pancreatic ductal adenocarcinoma	EV release of KRAS^G12D^/STAT3-dependent FAO	M2 polarization	[115]
Colorectal cancer	Exosomal miR-145/HDAC11 downregulation	M2 polarization. Tumor progression	[116]
Liver metastasis of colorectal cancer	Exosomal miRNAs/CXCL12/CXCR4/PTEN/PI3K/Akt	M2 polarization. EMT and metastasis	[117]
Colorectal cancer	PKM2/NF-κB p65–mediated CCL2 gene expression	Recruitment of macrophages	[118]
Melanoma	Acidification of TME induces GPCR-dependent expression of ICER in TAMs	M2 polarization	[119]
Lung carcinoma and melanoma	Anaerobic glycolysis/Lactate/VEGF-A and Arginase-1	M2 polarization and tumor growth	[120]
Melanoma	Hypoxia-induced HMGB1 and RAGE-dependent IL-10 production	M2-like TAM polarization. Tumor growth and metastasis	[121]
Nonsmall cell lung cancer	NOX4, ROS/PI3K and cytokine production (CCL7, IL-8, CSF-1, VEGF-C)	Macrophage recruitment, M2 polarization and tumor growth	[122]
Renal cell carcinoma	CCL2	Macrophage infiltration. Tumor growth and angiogenesis.	[123]
Esophageal squamous cell carcinoma	CCL2-CCR2 axis and PD-1 signaling pathway	Immune evasion	[124]

5-LOX: 5-lipoxygenase; C/EBPα: CCAAT/enhancer-binding protein alpha; CCL2: CC-chemokine-ligand-2; CCL7: CC-chemokine-ligand-7; CCL8: CC-chemokine-ligand-8; CCR2: CC-chemokine-receptor-2; CSF-1: colony stimulating factor 1; CXCLs: C-X-C motif chemokine ligands; EMT: epithelial-to-mesenchymal transition; FABP4: fatty acid-binding protein 4; FAO: fatty acid oxidation; FASN: fatty acid synthase; FOXP1: forkhead box P1; gp130: glycoprotein 130; GPCR: G-protein-coupled receptor; HDAC11: histone deacetylase 11; HMGB1: high-mobility group box 1; HSL: hormone-sensitive lipase; IL-10: interleukin-10; IL-4: interleukin-4; IL-6: interleukin-6; IL-8: interleukin-8; LPL: lipoprotein lipase; MMPs: matrix metalloproteinases; NF-κB: nuclear factor kappa B; NOX4: NADPH oxidase 4; Nrp-1: neuropilin-1; PAI-1: plasminogen activator inhibitor 1; PI3K: phosphoinositide 3-kinase; PKM2: pyruvate kinase M2; PLOD2: procollagen-lysine,2-oxoglutarate 5-dioxygenase 2; RAGE: receptor for advanced glycation end products; ROS: reactive oxygen species; SOCS3: suppressor of cytokine signaling 3; STAT3: signal transducer and activator of transcription 3; VEGF: vascular endothelial growth factor; ZEB1: zinc finger E-box binding homeobox 1.

**Table 4:** Antioxidants preventing cancer associated with obesity and systemic inflammation.

Drug	Mechanism	Cancer Type	Refs.
Curcumin and Salsalate	IL-1β, IL-6, Akt, NF-κB	Colon cancer	[163]
Curcumin and Salsalate	PI3K/Akt/mTOR/NF-κB/Wnt cytokines	Colorectal cancer	[164]
Curcumin	AMPK, COX-2, NF-κB, TNF, IL-6, adiponectin, leptin	Colonic preneoplastic lesions	[165]
Curcumin	Apoptosis, oxidation-induced DNA damage and DNA repair	Colorectal cancer	[166]
Epigallocatechin gallate	Protein levels of IGF-IR, p- GSK3β, beta-catenin, cyclooxygenase-2 and cyclin D1 Serum levels of IGF-I, insulin, triglyceride, cholesterol, and leptin	Colonic premalignant lesions	[167]
Silibinin	Obesity-induced cancer cells growth, ROS, lipogenesis, MMP-9 and cancer cell invasion FASN, IL-6, IL-1β and Erk	Liver Cancer	[168]
Naringenin	Adiposity and inflammation AMPK	Breast cancer	[169]
Taxifolin	Adipocyte, HGF, SOS1, Grb2, CCL2, macrophages	Breast cancer	[170]
Resveratrol	Adipocyte hypertrophy macrophage infiltration COX-2, PPARγ	Breast cancer	[171]
Resveratrol	Proliferation of cancer cells AMPK, Akt	Breast cancer	[172]

AMPK: AMP-activated protein kinase; CCL2: CC-chemokine-ligand-2; COX-2: cyclooxygenase-2; FASN: fatty acid synthase; Grb2: growth factor receptor bound protein 2; GSK3β: glycogen synthase kinase-3 beta; HGF: hepatocyte growth factor; IGF-I: insulin-like growth factor I; IGF-IR: insulin-like growth factor I receptor; IL-1β: interleukin-1β; IL-6: interleukin-6; MMP-9: matrix metalloproteinase-9; mTOR: mammalian target of rapamycin; NF-κB: nuclear factor kappa B; PI3K: phosphoinositide 3-kinase; PPARγ: peroxisome proliferator-activated receptor gamma; ROS: reactive oxygen species; TNF: tumor necrosis factor.

**Table 5:** Antioxidant effects in the tumor microenvironment.

Drug	Mechanism	Cancer Type	Refs.
Taxifolin	Adipocyte, HGF, SOS1, Grb2, CCL2, macrophages	Breast cancer	[170]
Resveratrol	Adipocyte hypertrophy Macrophage infiltration COX-2, PPARγ	Breast cancer	[171]
Vitamin E succinate	Vascular endothelial growth factor A, IL-10, and M2-like phenotype of tumor-associated macrophages	Colon cancer	[173]
Genistein	Reverts oxidative stress-induced cancer CD133, CD44, and β-catenin	Colon cancer	[174]
Berberine	EGFR-ERK TNF-α and IL-6	Colitis-associated tumorigenesis	[175]
Berberine	IL-1β, TNF, NF-κB	Intestinal carcinogenesis	[176]
Berberine	IL-6, IL-1β, COX-2 and TNF-α Microbiota STAT3, JNK and β-catenin	Colitis-associated carcinogenesis	[177]
Berberine	TNF-α and IL-6	Breast cancer	[178]
Epigallocatechin-3-gallate	Adipogenesis, STAT3	Breast cancer	[179]
Epigallocatechin-3-gallate	Prevents cancer to TME effects. CCL2, CCL5, IL-1β and IL-6 COX2, HIF-1α, VEGFα, and PD-L1 Smad2 and NF-κB	Breast cancer	[180]
Epigallocatechin-3-gallate	CD44	Prostate cancer	[181]
Quercetin and doxorubicin	CD44	Liver cancer	[182]
Diallyl trisulfide	S100A8/A9, serum amyloid A (SAA), IL-6, fibronectin, MRP8, myeloperoxidase (MPO) and TLR4-Myd88	Melanoma and lung metastasis	[183]
Nicaraven	CXCL10, SDF-1, IL-2 and MIP-2 MAPK	Lung carcinoma	[184]

CCL2: CC-chemokine-ligand-2; COX-2: cyclooxygenase-2; CCL5:CC-chemokine-ligand-5; CXCL10: C-X-C motif chemokine ligand-10; EGFR: epidermal growth factor receptor; Grb2: growth factor receptor bound protein 2; HGF: hepatocyte growth factor; HIF-1α: hypoxia-inducible factor 1 subunit alpha; IL-10: interleukin-10; IL-1β: interleukin-1β; IL-6: interleukin-6; JNK: c-Jun N-terminal kinase; MAPK: mitogen-activated protein kinase; MIP-2: macrophage inflammatory protein-2; MPO: myeloperoxidase; MRP8: myeloid-related protein 8; Myd88: myeloid differentiation primary response 88; NF-κB: nuclear factor kappa B; PD-L1: programmed death ligand 1; PPARγ: peroxisome proliferator-activated receptor gamma; SAA: serum amyloid A; SDF-1: stromal cell-derived factor 1; Smad2: small mother against decapentaplegic 2; STAT3: signal transducer and activator of transcription 3; TLR4: toll like receptor 4; TNF-α: tumor necrosis factor-alpha; VEGFα: vascular endothelial growth factor alpha.

## Data Availability

All the data are contained within the article.
